# Climate Change and Trophic Response of the Antarctic Bottom Fauna

**DOI:** 10.1371/journal.pone.0004385

**Published:** 2009-02-05

**Authors:** Richard B. Aronson, Ryan M. Moody, Linda C. Ivany, Daniel B. Blake, John E. Werner, Alexander Glass

**Affiliations:** 1 Department of Biological Sciences, Florida Institute of Technology, Melbourne, Florida, United States of America; 2 Dauphin Island Sea Lab, Dauphin Island, Alabama, United States of America; 3 Department of Marine Sciences, University of South Alabama, Mobile, Alabama, United States of America; 4 Department of Earth Sciences, Syracuse University, Syracuse, New York, United States of America; 5 Department of Geology, University of Illinois, Urbana, Illinois, United States of America; Monterey Bay Aquarium Research Institute, United States of America

## Abstract

**Background:**

As Earth warms, temperate and subpolar marine species will increasingly shift their geographic ranges poleward. The endemic shelf fauna of Antarctica is especially vulnerable to climate-mediated biological invasions because cold temperatures currently exclude the durophagous (shell-breaking) predators that structure shallow-benthic communities elsewhere.

**Methodology/Principal Findings:**

We used the Eocene fossil record from Seymour Island, Antarctic Peninsula, to project specifically how global warming will reorganize the nearshore benthos of Antarctica. A long-term cooling trend, which began with a sharp temperature drop ∼41 Ma (million years ago), eliminated durophagous predators—teleosts (modern bony fish), decapod crustaceans (crabs and lobsters) and almost all neoselachian elasmobranchs (modern sharks and rays)—from Antarctic nearshore waters after the Eocene. Even prior to those extinctions, durophagous predators became less active as coastal sea temperatures declined from 41 Ma to the end of the Eocene, ∼33.5 Ma. In response, dense populations of suspension-feeding ophiuroids and crinoids abruptly appeared. Dense aggregations of brachiopods transcended the cooling event with no apparent change in predation pressure, nor were there changes in the frequency of shell-drilling predation on venerid bivalves.

**Conclusions/Significance:**

Rapid warming in the Southern Ocean is now removing the physiological barriers to shell-breaking predators, and crabs are returning to the Antarctic Peninsula. Over the coming decades to centuries, we predict a rapid reversal of the Eocene trends. Increasing predation will reduce or eliminate extant dense populations of suspension-feeding echinoderms from nearshore habitats along the Peninsula while brachiopods will continue to form large populations, and the intensity of shell-drilling predation on infaunal bivalves will not change appreciably. In time the ecological effects of global warming could spread to other portions of the Antarctic coast. The differential responses of faunal components will reduce the endemic character of Antarctic subtidal communities, homogenizing them with nearshore communities at lower latitudes.

## Introduction

Polar marine organisms are physiologically adapted to cold-water conditions. Being cold-stenothermal places them at great risk from the direct effects of global warming [1]. Warming seas are also promoting biological invasions of polar marine ecosystems, with potentially catastrophic consequences [2]–[4].

The endemic character of the Antarctic nearshore fauna and its uniquely truncated trophic structure [5]–[7] are products of climatic cooling that began in the Eocene and led to the growth of an ice sheet on Antarctica at the Eocene–Oligocene boundary ∼33.5 Ma (million years ago) [8], [9]. The Antarctic climate of the early Eocene was temperate by today's standards. Shallow-subtidal communities contained the functionally modern durophagous (shell-breaking) teleostean fish, decapod crustaceans, and neoselachian sharks and rays that are typical of Cenozoic nearshore faunas worldwide. The long-term cooling trend that led to the polar climate of today began with a cooling step ∼41 Ma during the middle Eocene. At that time coastal sea temperatures dropped by as much as 10°C over a period of several million years [10]. Durophagous predators persisted in Antarctica until at least the end of the Eocene, but they were far less active after 41 Ma [2], [11]. They became extinct as temperatures declined further, although the timing of those post-Eocene extinctions is uncertain.

Concomitant with the sharp reduction in activity and eventual loss of the durophagous predators, all of which are products of post-Paleozoic evolutionary radiations [12], [13], benthic communities in Antarctica regressed to an archaic state. The top predators of the living Antarctic benthos are now asteroids, nemertean worms, and other slow-moving invertebrates of a Paleozoic functional grade that cannot break hard-shelled prey. As early as the middle to late Eocene, declining predation pressure released epifaunal suspension-feeding invertebrates from predation pressure. They grew in dense populations, forming communities of a type common in nearshore environments during the Paleozoic [11].

We used the fossil record preserved in the La Meseta Formation at Seymour Island, Antarctic Peninsula, to track the response of ecologically significant taxonomic and functional groups to the Eocene cooling event and decline in predation pressure. Here we draw on those paleontological patterns to predict how the current episode of global warming will reverse ecological trends that began in the Eocene. Climate change is already facilitating predatory reinvasions of the Antarctic Peninsula [2], [4], and cascading trophic effects are likely to occur in nearshore communities on a decadal to centennial time scale.

### Geology of the La Meseta Formation

The geologic setting, stratigraphy, depositional environment, and age of the Eocene La Meseta Formation at Seymour Island are reviewed in detail elsewhere [10] and summarized here. Seymour Island (64°15′S, 56°45′W) lies along a southwest–northeast axis, approximately 100 km southeast of the tip of the Antarctic Peninsula. The La Meseta Formation is the richest marine deposit in terms of macrofossils known from Antarctica, although there are numerous intervals in which fossils are rare or absent. The geology, geochemistry, and diverse composition of the fossil assemblages clearly indicate that the faunas inhabited a nearshore, shallow-water environment under fully marine conditions.

Sadler [14] divided the formation into seven discrete lithologic units, or Telms. (Telm is an acronym for “Tertiary Eocene La Meseta.”) Although there have been subsequent refinements of the stratigraphy [15], [16], for convenience we follow Ivany et al. [10] in retaining the Telm designations. The oldest unit, Telm 1, is represented by two small exposures at the margins of the La Meseta outcrop belt. The age of Telm 1 is poorly constrained. Telms 2–7, which are the focus of this paper, constitute the remainder of the La Meseta exposures. Telms 2–7 span most of the Eocene, from ∼54 Ma in the early Eocene to the Eocene–Oligocene boundary at ∼33.5 Ma. The pre-cooling interval, 55–41 Ma, is represented by Telms 2–5 and the lower two-thirds of Telm 6; the interval that includes the cooling event and its aftermath, 41–33.5 Ma, is represented by the upper third of Telm 6 and all of Telm 7 [10].

## Methods

Field expeditions to Seymour Island in 1986, 1994, 2000, 2001, and 2003 provided the opportunity to amass substantial collections of fossil marine invertebrates from Telms 2–7. Sites within the La Meseta Formation were plotted on a GPS-based, 1∶10000 topographic map of the island prepared in 1995 by the United States Geological Survey (USGS 64056-T5-TM-010). The sites were assigned to stratigraphic intervals based on our own mapping work and other sources [14], [17]. Fossil collections were enumerated, measured, and treated statistically as described in the Results section.

## Results

### Echinoderms and Brachiopods

**Figure 1:**
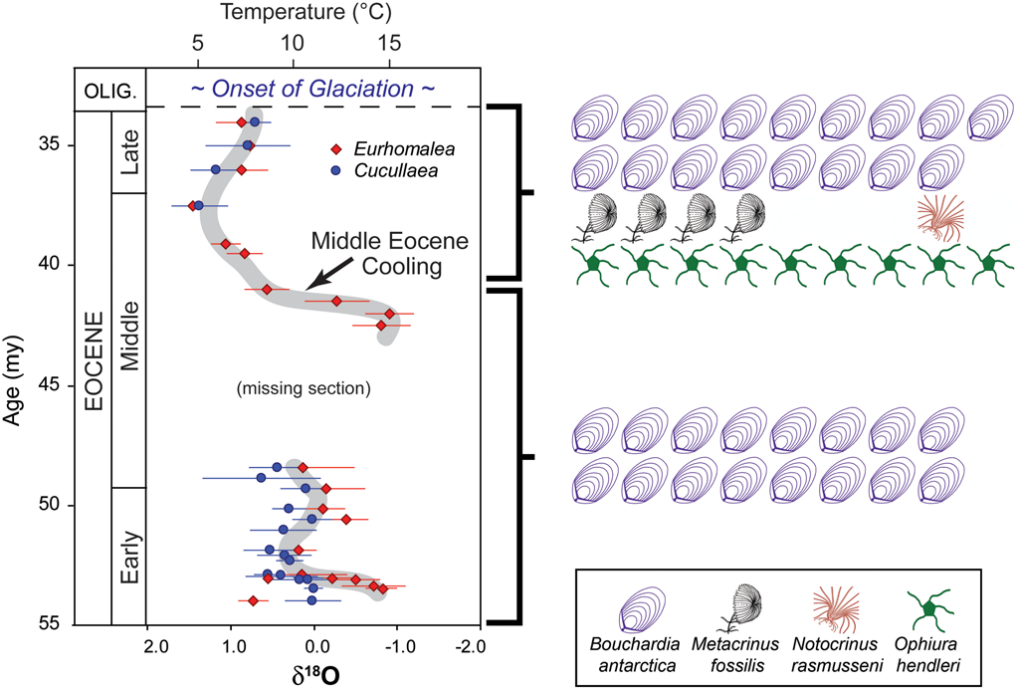
Distribution of epifaunal suspension-feeders in Telms 2–7 of the La Meseta Formation at Seymour Island, below and above the cooling event at 41 Ma. Graph on the left shows the Eocene paleotemperature curve derived from the La Meseta Formation at Seymour Island, based on mean oxygen-isotope values of shell material from two bivalve genera. Error bars represent standard deviations. Ages of horizons and the inferred presence of a middle Eocene unconformity are based on strontium-isotope stratigraphy (redrawn from [10]). Icons on the right denote dense, autochthonous or parautochthonous fossil concentrations that represent abundant paleopopulations of rhynchonelliform brachiopods, *Bouchardia antarctica*; stalked crinoids, *Metacrinus fossilis*; unstalked (comatulid) crinoids, *Notocrinus rasmusseni*; and ophiuroids, *Ophiura hendleri*. Paleontological data are based on surveys conducted in 1986, 1994, 2000, and 2001.

The stratigraphic distribution of autochthonous concentrations of articulated fossil echinoderms in the nearshore, shallow-marine facies that comprise the Eocene La Meseta Formation at Seymour Island shows that dense populations of ophiuroids (*Ophiura hendleri*) and crinoids (the stalked crinoid *Metacrinus fossilis* and the unstalked *Notocrinus rasmusseni*) flourished on soft substrata after the 41-Ma cooling event, but not before (Fig. 1; Table 1). Because ophiuroids and crinoids are vulnerable to both lethal and sublethal durophagy [18]–[20], their high abundances and low levels of sublethal damage (i.e., regenerating arms) are strong evidence for low predation pressure in these post-cooling populations [11].

**Table 1:** Occurrences of fossil assemblages representing dense populations of epifaunal suspension-feeders in the Telm units of the La Meseta Formation.

	Echinoderms			Brachiopods
Telm	*Ophiura*	*Metacrinus*	*Notocrinus*	*Bouchardia*
7	8	4	1	12
Upper 6†	1	0	0	5
Lower 6‡	0	0	0	0
5	0	0	0	7
4	0	0	0	6
3	0	0	0	3
2	0	0	0	0
1	0	0	0	2

† Upper third of Telm 6, from ∼41 to ∼39 Ma.

‡ Lower two-thirds of Telm 6, before the cooling event at ∼41 Ma.

An alternative hypothesis is that low-salinity conditions excluded dense ophiuroid and crinoid populations from the pre-cooling interval. This explanation can be eliminated for two reasons. First, other echinoderms, including asteroids and irregular echinoids, occur throughout the La Meseta Formation. Echinoderms have no excretory organs and as a result are strictly stenohaline; hyposaline conditions would have eliminated all echinoderms, not just dense populations of ophiuroids and crinoids. Second, geochemical data point to normal marine salinities during deposition of the fossiliferous intervals [10].

The presence of articulated asteroids from the basal Telm 1 to the uppermost Telm 7 of the La Meseta Formation provides a taphonomic control. Asteroids, like ophiuroids and crinoids, are susceptible to post-mortem disarticulation. If ophiuroids and crinoids had lived in dense populations prior to the cooling event, they would have been as readily preserved as the asteroids in the lower units.

Freed from the constraints of durophagy, dense populations of ophiuroids and sessile suspension-feeders cover vast areas of the soft-sediment seafloor in Antarctic shelf environments to this day [7], [21]. *Ophionotus victoriae*, the ophiuroid species that commonly forms modern dense populations, is a low-energy generalist, feeding on zooplankton in the water column as well as living and dead organic matter on the benthos [22]. Comatulid (unstalked) crinoids, the only crinoids which currently inhabit nearshore environments anywhere [18], are generally restricted in shallow Antarctic waters to rocky substrata and the stalks and branches of sessile invertebrates [7], but they have also been recorded occasionally in dense aggregations on unconsolidated substrata in <100 m depth [23]. The comatulids are suspension-feeders and, like the Antarctic ophiuroids, are adapted to surviving on low energy budgets [22]. Stalked crinoids were eliminated from Antarctic shallow waters after the Eocene, and today the genus *Metacrinus*, like all taxa of stalked crinoids worldwide, is confined to the deep sea [24].

In contrast to the ophiuroid and crinoid assemblages, spatially discrete concentrations of the rhynchonelliform brachiopod *Bouchardia antarctica* (Terebratellidae) occurred in the shallow-marine, soft-substratum paleoenvironments of the La Meseta Formation both before and after the 41-Ma temperature drop and associated decline in predation pressure (Fig. 1). The temporal distributions of brachiopod and echinoderm assemblages in Telms 2–7 differed significantly from each other across the cooling event (all echinoderm assemblages pooled; χ^2^ = 10.291, *df* = 1, *P* = 0.001). Incorporating data from Telm 1 (Table 1) does not alter the strength of the observed pattern (χ^2^ = 11.381, *df* = 1, *P* = 0.001).

*Bouchardia antarctica* is the only species in the formation found in dense brachiopod “nests,” which consist of tens to hundreds of specimens distributed over a square meter or less. Despite the possibility of centennial- to millennial-scale time-averaging, which has been observed in shell assemblages of a modern congener in Brazil, *B. rosea* [25], the abundance of articulated *B. antarctica* in good taphonomic condition in many of the Eocene brachiopod nests at Seymour Island suggests they formed dense living populations. Brachiopods are suspension-feeders, and *B. rosea* is epifaunal to semi-infaunal [26]; a similar life habit is inferred for *B. antarctica*. Conditions in the late Eocene following the cooling event may have favored dense populations of *B. antarctica* (Table 1), but brachiopod nests do not show the sudden post-cooling increase observed for dense echinoderm populations.

### Morphometric Analysis of Brachiopod Shells

If predation pressure on brachiopods did not change appreciably across the 41-Ma cooling event, then the defensive attributes of their shells should not have changed either. Shell morphologies of *Bouchardia antarctica* from two sites in Telm 5 (B1 and 94-18; pre-cooling) and two in Telm 7 (94-11 and 94-12; post-cooling) were compared using Principal Components Analysis (PCA). Only well-preserved, undistorted, articulated valve-pairs were used. Four linear shell metrics, which could be measured precisely in the fossil specimens, were recorded for each individual: length of the pedicle valve, length of the brachial valve, width of the brachial valve, and shell height. Shell height, the maximum thickness of the valve-pair in the closed position, is a measure of shell inflation. Shell heights of gaped specimens were calculated by regressing shell height on pedicle valve length for closed specimens from the same site. Each variable was measured to the nearest 0.01 mm using digital calipers. Total sample sizes were *n* = 69 for Telm 5 and *n* = 56 for Telm 7.

We used PCA to examine the proportion of the total variance in shell morphology distributed among sites and time intervals (Telms). The data were 1/*x*-transformed prior to analysis to satisfy the requirement that samples be drawn from a multivariate, normal distribution. (The commonly used logarithmic transformation did not normalize the data.) Significant eigenvector loadings were identified using Pearson correlation analysis between the independent variables and their corresponding loadings for each principal component (PC).

**Figure 2:**
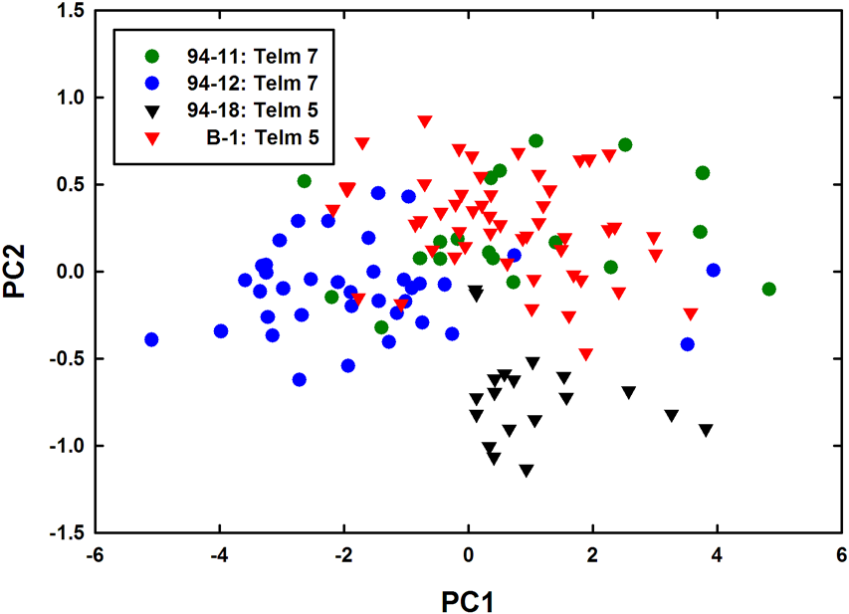
Morphometric analysis of *Bouchardia antarctica* shells from before and after the 41-Ma cooling event. Shell-morphology scores for the first two principal components explain 97.7% of the among-site variability. PC1 describes variability in size and PC2 represents variability in shape. Sites B-1 and 94-18 predate the cooling event, whereas sites 94-11 and 94-12 postdate the event.

Two PCs extracted from the correlation matrix explained 97.7% of the variability in the data set (Fig. 2, Table 2). PC1, which accounted for 92.8% of the total variance, yielded highly negative loadings for all independent variables, suggesting that it represents variability in shell size; specimens with high PC1 scores had small shells, whereas those with low scores had large shells. PC2 accounted for 4.8% of the total variance; it yielded a significantly positive loading for shell height and a significantly negative loading for shell width. We interpret PC2 as describing variability in shell shape, essentially independent of shell size. Specimens with low PC2 scores had wide, flat shells, whereas those with high PC2 scores had inflated, narrow shells.

**Table 2:** Eigenvector loadings and eigenvalues for PCA of four linear shell measurements of *Bouchardia*.

Results	Original variable	PC1	PC2
**Eigenvector loadings**	Pedicle valve length	−0.513*	0.083
	Brachial valve length	−0.511*	−0.013
	Shell height	−0.489*	0.664*
	Shell width	−0.486*	−0.743*
**Eigenvalues (%)**		92.8	4.8

Loadings denoted with an asterisk (^*^) are significantly correlated with the original independent variables (Pearson correlations; *P*≤0.0010; Bonferroni-corrected α_adj_ = 0.00625 for 8 tests).

A nested Analysis of Variance (ANOVA) was performed on the PC2 scores, with Telm as the fixed factor and Site nested within Telm. There was no significant effect of Telm (*F* = 0.28, *df* = 1, 121; *P* = 0.649). There was, however, a significant Site effect (*F* = 91.87; *df* = 2, 121; *P*<0.0005). A nested ANOVA on the PC1 scores also yielded a significant Site effect (*F* = 16.47; *df* = 2, 121; *P*<0.0005) but no significant Telm effect (*F* = 1.19, *df* = 1, 121; *P* = 0.390). In summary, the morphometric analysis showed no consistent changes in shell shape or size associated with either the temperature drop or declining predation pressure.

### Shell-Breaking and Shell-Drilling Predation

None of the hundreds of *B. antarctica* collected from pre- and post-cooling horizons displayed evidence of sublethal shell breaks (repaired cracks). The absence of sublethal damage could indicate either very low or very high frequencies of lethal predation [11], [27]. In aggregate, however, the distributional, morphometric, and shell-repair data argue that *Bouchardia* populations did not experience significant shell-breaking predation before or after the 41-Ma cooling event.

Brachiopods, like bivalves, are potentially susceptible to predation by shell-drilling gastropods. We compared evidence of drilling predation between nests of *Bouchardia* and dense aggregations of venerid bivalves, *Eurhomalea* spp. *Eurhomalea* is the most abundant genus of infaunal bivalves in the La Meseta Formation. Of 93 well-preserved, articulated pairs of *Bouchardia* valves recovered from 5 dense aggregations before the 41-Ma cooling event, and 116 pairs from 5 dense aggregations after the event, none (0%) had a borehole. In contrast, naticid boreholes occurred in 4.9% of well-preserved, articulated valve-pairs of *Eurhomalea* spp. (Veneridae) in dense fossil aggregations from before the cooling event, and in 5.7% of *Eurhomalea* valve-pairs in dense aggregations from after 41 Ma (Table 3; χ^2^ = 0.796, *df* = 1, *P* = 0.372). Almost all the boreholes in *Eurhomalea* from both intervals—154 out of 157, or 98%—were complete, indicating that the preponderance of attacks were lethal both before and after the cooling event. Inclusion of disarticulated valves in the calculations yielded similar results, with drilling frequencies of 5.9% before the cooling event and 6.0% after 41 Ma (Table 3; χ^2^ = 0.408, *df* = 1, *P* = 0.523). Combining articulated and disarticulated *Eurhomalea* valves, 183 of 188 drillholes, or 97.3%, were lethal. No valve-pair or disarticulated valve had more than one borehole.

**Table 3:** Frequencies of naticid drillholes in *Eurhomalea* spp. bivalves from the La Meseta Formation.

Valves analyzed	Interval	*N*	Complete	Incomplete	Drilled (%)
**Paired**	Post-cooling	1750	96	3	5.66
	Pre-cooling	1246	61	0	4.90
**Paired plus disarticulated**	Post-cooling	1830	106	4	6.01
	Pre-cooling	1329	77	1	5.87

Bivalves were analyzed from 9 pre-cooling sites and 8 post-cooling sites. For the samples of paired plus disarticulated valves, the number of disarticulated valves was divided by 2 to calculate the number of individuals, *N*.

Declining temperatures in the Eocene thus did not appreciably alter the frequency of shell-drilling predation by infaunal naticid gastropods on the infaunal *Eurhomalea*. The epifaunal/semi-infaunal *Bouchardia* were not affected by drilling gastropods either before or after the cooling event. The dense aggregations of *Eurhomalea* and *Bouchardia* were found separately, so the living populations may have occupied different microhabitats. Our results are nevertheless consistent with the observed low levels of drilling predation in modern Brazilian populations of *B. rosea*, with higher frequencies in co-occurring venerids and other infaunal bivalves [26]. Thus, predatory gastropods may have been physically segregated from *B. antarctica* on a small spatial scale, or they may have co-occurred but avoided eating them.

The numerically dominant gastropods of the La Meseta Formation are semi-infaunal species in the families Naticidae and Struthiolariidae. Like *Bouchardia*, the naticids show little morphological variation and no taxonomic change across the 41-Ma cooling event. The struthiolariids exhibit a species-replacement sequence in which they become smaller and thinner-shelled upsection, but apparently not in association with the cooling event [17].

Rhynchonelliform brachiopods at all latitudes, including *Bouchardia*, are low-energy, sessile suspension-feeders that today survive under both oligotrophic and eutrophic conditions [28]–[30]. Fossil evidence suggests they were unaffected or weakly affected by post-Paleozoic secular trends in shell-drilling and shell-breaking predation [31], [32]. *B. antarctica* apparently tolerated a broad range of ecological conditions over Eocene time, including substantial variations in productivity and the activity of predators [10], [11], which would explain why dense aggregations are found throughout the La Meseta Formation. Today, aggregations of the rhynchonelliform *Liothyrella uva* are prominent constituents of the nearshore benthos off the Antarctic Peninsula, but unlike *Bouchardia* the aggregations occur on rocky bottoms rather than soft substrata [7], [33].

## Discussion

Sea-surface temperatures off the western Antarctic Peninsula (WAP) have risen by 1°C in the last 50 years, making that area one of the fastest-warming regions of the World Ocean [34]. Up to this point, brachyuran and anomuran crabs (and other reptant decapods) have been excluded from Antarctic shelf environments by an unusual physiological constraint: they are unable to down-regulate the magnesium ions they naturally take up from seawater by diffusion. Lower temperatures decrease their scope for aerobic activity, and the added narcotic effect of Mg^2+^ is lethal at temperatures below ∼1°C [35]. Predatory crabs are now reinvading WAP: their larvae cross the Polar Front in warm-core rings and in the ballast-water released by ships [36]. Furthermore, adult populations of lithodids (anomuran king crabs) have recently been discovered in the deeper waters of the continental slope off the WAP, which are slightly warmer at 1–2°C [4].

Continued warming of shallow waters and break-up of the ice shelves will likely increase water-column productivity and prolong the growing season for incoming crab larvae [37], [38]. Warmer sea temperatures will also permit lithodids from the slope to move onto the shelf and establish viable predatory populations. Durophagous fish from lower latitudes may be able to invade as well [2].

Running the Eocene cooling event in rapid-reverse, we predict the trajectories of three taxonomic and functional components of the benthic fauna. First, increasing predation pressure associated with climatic warming and introduction of exotic taxa over the next decades to centuries will reduce or eliminate the dense populations of epifaunal ophiuroids and crinoids that currently flourish in Antarctic shelf environments. Second, epifaunal brachiopods, in contrast, will not decline precipitously in the face of increasing durophagy. Supporting this second prediction, rhynchonelliform brachiopods are currently prominent constituents of the rocky-subtidal epifauna at subpolar, temperate and tropical latitudes in the Southern Hemisphere [29], [33]. Third, the intensity of shell-drilling predation on infaunal bivalves will not change appreciably. These trophic effects will occur first off the WAP and could eventually spread to other coastal areas of Antarctica.

Even if rising temperatures were to eliminate *Ophionotus victoriae* from subtidal habitats in Antarctica prior to any predation effects, its taxonomic and ecological equivalent from the subantarctic, *O. hexactis* [22], would likely expand poleward. Subantarctic brachiopods such as *Magellania* spp. potentially could replace *Liothyrella uva* [30], [39]. The differential effects of increasing durophagous predation would play out on replacement taxa in an analogous fashion. The effects of ocean acidification on ophiuroids and other faunal elements remain uncertain [1], [40], so it would be premature to speculate about how declining pH might affect predator–prey relationships in the Antarctic benthos.

The Antarctic shelf fauna expanded and contracted during the Pleistocene glacial cycles [41]. It is possible that durophagous predators appeared during warm periods, but this is by no means certain given the poor Pleistocene record in Antarctica. Today's situation differs from the Pleistocene interglacials and the mid-Holocene climatic optimum in its artificially rapid rate of change [1], [34] and the fact that humans are now introducing durophagous predators from as far away as the sub-Arctic [4]. Mitigating the direct introduction of exotic predators will require strengthening the Antarctic Treaty to tighten controls on rapidly expanding ship traffic in Antarctica. The Treaty, however, cannot address global warming. Without a comprehensive, international commitment to control greenhouse-gas emissions, climate change will promote biotic invasions and destroy the endemic character of the Antarctic shelf fauna, homogenizing it taxonomically and functionally with nearshore faunas elsewhere.
